# A Moderate Supplementation of Native Whey Protein Promotes Better Muscle Training and Recovery Adaptations Than Standard Whey Protein – A 12-Week Electrical Stimulation and Plyometrics Training Study

**DOI:** 10.3389/fphys.2018.01312

**Published:** 2018-09-19

**Authors:** Sebastian Garcia-Vicencio, Sébastien Ratel, Céline Gryson, Aurélie Masgrau, Enzo Piponnier, Jacqueline Brasy, Pascale Le Ruyet, Marion Bucas, Nicolas Barachon, Victoire Visseaux, Yann Connan, Florence Montel, Clément Lahaye, Yves Boirie, Vincent Martin

**Affiliations:** ^1^AME2P, CRNH Auvergne, University Clermont Auvergne, Clermont-Ferrand, France; ^2^Lactalis Research & Development, Retiers, France; ^3^Lactalis Ingredients, Bourgbarré, France; ^4^Lactalis Ingredients USA, Buffalo, NY, United States; ^5^Clinical Nutrition, Clermont-Ferrand University Hospital, Clermont-Ferrand, France; ^6^Human Nutrition Unit, INRA, UNH, CRNH Auvergne, University Clermont Auvergne, Clermont-Ferrand, France

**Keywords:** soluble milk protein, force, electrostimulation, training, recovery, neuromuscular properties, fatigue

## Abstract

The purpose of this study was to assess if native whey protein (NW) supplementation could promote recovery and training adaptations after an electrostimulation (ES) training program combined to plyometrics training. Participants were allocated into three groups, supplemented 5 days/week, either with 15 g of carbohydrates + 15 g of NW (*n* = 17), 15 g of carbohydrates + 15 g of standard whey protein (SW; *n* = 15), or placebo (PLA; 30 g of carbohydrates; *n* = 10), while undergoing a 12-week ES training program of the knee extensors. Concentric power (Pmax) was evaluated before, immediately after, as well as 30 min, 60 min, 24 h, and 48 h after the 1st, 4th and last ES training session. The maximal voluntary contraction torque (MVC), twitch amplitude, anatomical cross-sectional area (CSA) and maximal voluntary activation level (VA) were measured before (T0), and after 6 (T1) and 12 weeks of training (T2). *P*_max_ recovery kinetics differed between groups (*p* < 0.01). *P*_max_ started to recover at 30 min in NW, 24 h in SW and 48 h in PLA. Training adaptations also differed between groups: MVC increased between T0 and T2 in NW (+11.8%, *p* < 0.001) and SW (+7.1%, *p* < 0.05), but not PLA. Nevertheless, the adaptation kinetics differed: MVC increased in NW and SW between T0 and T1, but an additional gain was only observed between T1 and T2 in NW. VA declined at T1 and T2 in PLA (−3.9%, *p* < 0.05), at T2 in SW (−3.5%, *p* < 0.05), and was unchanged in NW. CSA increased, but did not differ between groups. These results suggest that NW could promote a faster recovery and neuromuscular adaptations after training than SW. However, the mechanisms underlying this effect remain to be identified.

## Introduction

During physical training, recovery is of utmost importance. Indeed, the recovery process alleviates the negative consequences of fatigue and promotes improvement in physical abilities. Together with training load, this is the core of physical training. The issue of recovery gets even more critical when the level of practice increases, since the frequency and duration of training are generally very high in elite athletes (Barnett, 2006). Some training modalities, such as long duration exercise bouts, eccentric exercise or electrostimulation (ES) training may also generate particular recovery issues, since they generate a high physiological stress on the motor units. For instance, it has been demonstrated that both eccentric and ES exercise bouts can generate muscle damage (Clarkson et al., 1986; Jubeau et al., 2012), which triggers an inflammatory response. This inflammation is associated with a slow recovery, which can last several days (Nosaka and Sakamoto, 2001). In addition, ES training can lead to a state of overreaching at the end of an ES training program, translating into blunted training adaptations (Zory et al., 2010).

Therefore, several strategies have been designed to accelerate recovery following these forms of exercise. These strategies may include manual therapies (e.g., massage, stretching), physical therapies (e.g., water immersion, compression) and nutritional strategies (Barnett, 2006). In relation to nutritional strategies, several nutrients with a potential recovery-enhancing effect have been tested: antioxidants, vitamins, carbohydrates, branched-chain amino acids, proteins (Pasiakos et al., 2014; Sousa et al., 2014). Proteins, and specifically when their content is high in branched-chain amino acids, have the potential to stimulate protein synthesis and inhibit proteolysis (Zanchi et al., 2008; Nicastro et al., 2011). This combined influence on synthesis and breakdown could result in a positive net-protein balance, improved contractile function, thereby promoting recovery, especially after damaging exercise bouts such as ES. Protein quality, or their digestive behaviors like the speed of absorption, may also differentially modulate these effects. For instance, milk contains two protein fractions: whey proteins (soluble proteins) and caseins, with rapid and slow digestion rates, respectively, considered now as an independent factor of postprandial protein anabolism (Boirie et al., 1997; Dangin et al., 2001). Muscle protein synthesis has been shown to be greater with the consumption of whey proteins (SW) when compared with casein (Tang et al., 2009; Pennings et al., 2011), although one study also reported no difference (Reitelseder et al., 2011). This greater efficacy of SW is related to its higher content of leucine but also to the peak leucine concentration in plasma which appears faster in relation with the stimulation of muscle protein synthesis (Boirie et al., 1997; Pennings et al., 2011). Indeed, beyond its role in protein building, leucine ingestion results in the phosphorylation of intracellular proteins that are critical for mTOR signaling pathway, thereby leading to the stimulation of the initiation of muscle protein synthesis (Atherton et al., 2010). The quality and type of leucine rich milk proteins may also affect their recovery-enhancing effects. SW are traditionally extracted as concentrate or isolate from cheese whey, which is the by-product of cheese production. Recently, native whey protein isolate (NW), which contains 95% protein on dry basis, and is directly extracted from skimmed milk by physical separation using membrane technologies at low temperature, has been used to promote recovery after an immobilization (Martin et al., 2013; Verney et al., 2017), and to reduce training-induced fatigue in young (Babault et al., 2014) or elderly men (Gryson et al., 2014). NW supplementation induces a higher and faster peak value in blood leucine than SW after a single bout of strength training (Hamarsland et al., 2017a). However, this acute supplementation does not translate into an acute faster recovery of muscle function or increased muscle protein synthesis as compared to SW (Hamarsland et al., 2017a,b). It has nevertheless been suggested that beneficial effects of NW on recovery may be more evident over time, in the context of a long-term training study (Hamarsland et al., 2017b). Interestingly, long-term NW supplementation combined to physical training was shown to reduce exercise-induced fatigability during training as compared to casein (Babault et al., 2014) and milk proteins (Gryson et al., 2014).

Whether NW has greater recovery-enhancing effects than SW in the context of physical training, remains therefore unknown. Interestingly, we recently demonstrated that long-term NW supplementation promotes muscle mass recovery and oxidative enzymes activities during the recovery period after an immobilization in rats as compared to SW (Verney et al., 2017). In the context of physical training, this could potentially promote recovery, reduce the risk of overreaching and consequently, promote training adaptations. The purpose of this study was to test this hypothesis in the context of an ES training program combined with a SW or NW supplementation.

## Materials and Methods

### Participants

Forty-two young moderately active men [21.5 ± 3.2 years (mean ± SD)] volunteered to participate. To be included in the study they had to exercise less than 4 h/week, as assessed by the Global Physical Activity Questionnaire (Cleland et al., 2014). None of them was resistance-trained and all the participants had a recreational practice of sports. This study was approved by the local ethics committee (CPP Sud-Est VI; authorization number AU1153). All the participants were informed of the experimental procedures and gave their written informed consent before any testing was conducted, in accordance with the Declaration of Helsinki.

### Experimental Design

The study utilized an independent group design that was double-blind, randomized and placebo-controlled. Participants were randomly assigned in a block fashion, to one of three supplement groups; (1) NW (*n* = 17), (2) SW (*n* = 15), (3) iso-caloric carbohydrate placebo (PLA; *n* = 10). Initially, 18 participants were recruited in the NW and SW groups, and 12 in the PLA group. These sample size estimates were calculated from unpublished data on neuromuscular variables recovery after an ES session with a statistical power of 0.8. Six of these participants abandoned the study or did not attend all the testing sessions, such that their data were not considered in the statistical analysis (NW: *n* = 1; SW: *n* = 3; PLA; *n* = 2). The baseline characteristics of the groups are detailed in **Table 1**.

**Table 1:** Baseline characteristics.

Variable	NW	SW	PLA	ANOVA (*p*)
**Anthropometrics**				
Age (y)	21.4 ± 2.5	20.8 ± 3.3	22.2 ± 3.4	0.48
Body mass (kg)	74.0 ± 5.7	73.4 ± 3.5	74.4 ± 5.3	0.41
Height (m)	1.8 ± 0.1	1.8 ± 0.1	1.8 ± 0.1	0.96
BMI (kg/m^2^)	23.5 ± 1.6	23.5 ± 1.6	23.8 ± 1.5	0.83
Body fat (%)	13.4 ± 3.0	14.6 ± 2.7	13.6 ± 2.4	0.44
**Performance**				
Concentric power @ 180°/s (W)	561.5 ± 73.4	529.1 ± 84.7	566.8 ± 105.2	0.41
SJ power (W)	905.2 ± 96.2	893.5 ± 47.2	915.1 ± 72.7	0.77
MVC (N.m)	279.9 ± 64.2	273.5 ± 46.2	290.3 ± 64.4	0.76
Qtw, pot (N.m)	70.5 ± 12.6	70.1 ± 11.7	77.5 ± 11.7	0.24
VA (%)	91.4 ± 5.3	93.1 ± 3.1	93.0 ± 4.1	0.41
CSA (cm^2^)	38.1 ± 9.6	42.8 ± 9.9	50.9 ± 9.2	0.005
20 m Sprint (s)	3.2 ± 0.1	3.2 ± 0.1	3.2 ± 0.2	0.50
**Diet composition**				
Energy intake (kJ)	624.4 ± 141.8	587.4 ± 98.0	559.0 ± 113.1	0.41
Proteins (g)	119.4 ± 37.8	111.5 ± 22.0	97.5 ± 27.5	0.24
Proteins (g/kg)	1.60 ± 0.54	1.51 ± 0.29	1.31 ± 0.41	0.29
Carbohydrates (g)	257.3 ± 78.1	249.5 ± 65.3	272.6 ± 47.4	0.72
Fat (g)	115.7 ± 24.9	101.6 ± 24.5	88.1 ± 31.3	0.048

All the participants were supplemented 5 days/week during a 12-week training program (supplementation details are provided in section “Supplementation”). During the first 6 weeks, the participants performed 3 ES training sessions per week. From weeks 7 to 10, one ES training session was combined to 2 plyometrics training sessions per week to transfer the training adaptations into sport-specific movements [sprinting and jumping; (Malatesta et al., 2003)]. Finally, the training volume was reduced (1 ES-training session + 1 plyometrics training session) during weeks 11 and 12 to allow tapering (Zory et al., 2010).

**FIGURE 1:**
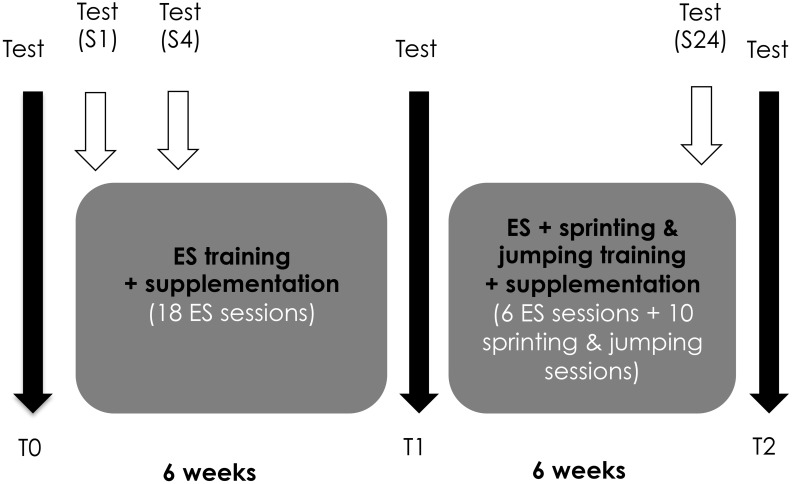
Overview of the experimental protocol. Three testing sessions were organized before (T0), and after 6 (T1) and 12 weeks (T2) of training to evaluate the training adaptations. The recovery kinetics of neuromuscular properties were evaluated after the 1st (S1), 4th (S4), and last (S24) electrostimulation (ES) sessions.

To evaluate training adaptations, three testing sessions were organized before (T0), and after 6 (T1) and 12 weeks (T2) of training (**Figure 1**). Anthropometrical characteristics, dimensions and neuromuscular properties of the knee extensor (KE) muscles and sprinting and jumping performances were the measured outcomes.

To describe the evolution of recovery capacities over the training program, the recovery kinetics of neuromuscular properties were evaluated after the 1st (S1), 4th (S4), and last (S24) ES sessions (**Figure 1**). The rationale for the choice of these time points was to get an overview of the recovery abilities at the beginning (S1) and end (S24) of the training process, and also when muscle damage and inflammation are maximal, i.e., after few training sessions (S4). Perceived muscle soreness, KE concentric torque and creatine kinase (CK) activity were measured before, immediately after and 30 min, 60 min, 24 h, and 48 h after the ES session.

These test sessions were preceded at baseline by a session dedicated to the clinical examination by a medical doctor and the familiarization with the experimental procedures. Each test session was organized at the same time of day (±1 h) to minimize the effects of diurnal variation. At each visit, participants were deemed fit for testing if they could confirm that they had not consumed medications/vitamins and had refrained from external exercise and alcohol during the preceding 24 h. Participants also refrained from consumption of nutritional supplements for the duration of the study period.

### Supplementation

Participants were supplemented with a 250-ml drink containing either 15 g of carbohydrates + 15 g of NW (Pronativ^®^), 15 g of carbohydrates + 15 g of standard whey protein from a cheese production process (SW group), or 30 g of carbohydrates for the PLA group (**Table 2**). This supplement was made from powder, diluted in water. The color, texture, and taste were similar to milk chocolate and were comparable and isocaloric between the different supplements. This suboptimal dose of protein was chosen, as recommended by Hamarsland et al. (2017a), to avoid a ceiling effect in muscle protein synthesis that would have hidden any potential difference between protein qualities (Churchward-Venne et al., 2014; Bukhari et al., 2015).

**Table 2:** Composition of the supplements.

	NW per serving (35 g + 250 ml water)	SW per serving (35 g + 250 ml water)	PLA per serving (35 g + 250 ml water)
Energy (kcal)	128.21	130.00	127.00
Proteins (g)	15.58	15.59	0.52
Carbohydrates (g)	15.60	14.60	30.49
Fibers (g)	0.78	0.78	0.78
Fat (g)	0.30	0.95	0.26

Pronativ^®^ (Lactalis Ingredients, Bourgbarré, France) is a 95% (on dry basis) soluble milk protein isolate. It is representative of native proteins in the non-casein phase of milk as it is an isolate of native whey proteins extracted directly from skimmed cow milk by a mild physical separation membrane process (Lactalis Ingredients, Bourgbarré, France), and not from a cheese process standard whey production. The amino acid compositions of the supplements are given in **Table 3**.

**Table 3:** Amino acids composition (g) for 35 g (serving) of standard whey (SW) and native whey (NW) proteins.

	NW	SW
	
	g/35g (per serving)	
Alanine	0.75	0.76
Arginine	0.48	0.4
Aspartic acid	1.78	1.6
Cystine	0.4	0.35
Glutamic acid	2.89	2.68
Glycine	0.32	0.28
Histidine	0.34	0.27
Isoleucine	0.93	1
Leucine	2.06	1.71
Lysine	1.62	1.36
Methionine	0.34	0.34
Phenylalanine	0.6	0.47
Proline	0.8	0.91
Serine	1.61	1.87
Threonine	0.82	1.07
Tryptophane	0.36	0.3
Tyrosine	0.64	0.49
Valine	0.86	0.88

Supplementation was given in a fasted state, 5 days per week at the same time of day, immediately after each training session (3/week; Monday, Wednesday, and Friday), and before breakfast, on non-training days (2/week; Tuesday and Thursday). No supplementation was given during the weekend days.

To check diet stability, participants were asked to record all energy-containing food and drinks intake in a diary on three consecutive days during the 1st, 6th, and 12th weeks of the training program. Dietary distribution of macronutrients and intake of total energy were analyzed following the recording period using the Nutrilog^®^ 2.10 software (Nutrilog^®^, Marans, France).

### Training Program

During the first 6 weeks, the participants trained on Monday, Wednesday, and Friday with ES at the same time of day. Forty bilateral isometric contractions of the KE were carried out during each ES session. During the stimulation, participants were seated on a bench with straps attached to the ankles to maintain the knee joint at 90°. Three 2-mm-thick self-adhesive electrodes were placed over each thigh. The positive electrodes, measuring 25 cm^2^ (5 cm × 5 cm), were placed as close as possible to the motor point of the *vastus lateralis* and *vastus medialis* muscles. The negative electrode, measuring 50 cm^2^ (10 cm × 5 cm), was placed 5–7 cm below the inguinal ligament. Rectangular-wave pulsed currents (75 Hz) lasting 400 μs were delivered by a battery-powered stimulator (Compex SP 2.0, DJO France, Mouguerre, France) with a rise time of 1.5 s, a steady tetanic stimulation time of 4 s, and a fall time of 0.75 s. Each stimulation was followed by a 20-s pause. Stimulation intensity was monitored online and was gradually increased throughout the training session to a level of maximally tolerated intensity, which varied as a function of the pain threshold of each subject. The average torque produced by ES was 70 ± 24% of the MVC.

During weeks 7–12, two ES sessions were replaced by sprinting and jumping training to transfer the ES-training adaptations into sport-specific movements, as recommended by Malatesta et al. (2003). These training sessions involved plyometric drills and short sprints (<25 m). The training load was progressively increased over the training period, except for the last 2 weeks, when the training volume was reduced to promote recovery and tapering.

### Evaluation of Training Adaptations

#### Anthropometrical Measurements

Body mass was measured to the nearest 0.1 kg using a calibrated scale. The positioning of the ultrasound probe was normalized for muscle dimensions. Thigh length was used as reference and measured using anatomical landmarks (Kubo et al., 2003).

#### Muscle Dimensions

A B-mode real-time ultra sound (US) scanner (Echo Blaster 128 CEXT-1Z, Telemed Ltd., Vilnius, Lithuania) with a linear array transducer (width: 59 mm; frequency range: 5–10 MHz) was used to obtain cross-sectional images of the KE muscles. Panoramic images (InVivo ScanNT 3.6 software, Medcom, Telemed Ltd., Lithuania) of the muscles were generated by moving the probe in the axial plane, with slow and continuous movement from the lateral to the medial side along a line marked on the skin. The anatomical cross-sectional area (CSA) of the *rectus femoris*, *vastus medialis*, and *vastus lateralis* muscles were manually traced off-line and summed to get an estimate of the KE CSA. Dimensions of the *vastus intermedius* muscle were difficult to determine in some individuals. Consequently, they were not reported here. All measurements were performed after the participants had laid in the supine position for at least 20 min to allow fluid shift to occur (Berg et al., 1993). Scans of the KE muscles were taken distally at 66% of the thigh length with the knees fully extended (Noorkoiv et al., 2010). The US probe was coated with a water-soluble transmission gel (Aquasonic, Parker Laboratory, Fairfield, NJ, United States) to improve acoustic coupling. Probe alignment was considered correct when aponeurosis could be delineated without interruption across the image. All US scans were performed by the same investigator.

Two consecutive scans were taken for each muscle and the average of the three measurements performed on each scan was used for the subsequent analysis. Images were analyzed off-line using the ImageJ software (Version 1.42, National Institutes of Health, Bethesda, MD, United States).

The intra- and inter-rater reliability of this evaluation is reported in **Table 4**. The reliability is good and consistent with previous report (Garcia-Vicencio et al., 2016).

**Table 4:** Intra- and inter-measurement reliability of the anatomical cross-sectional area of the knee extensor muscles.

		Intra-measurement reliability												Inter-measurement reliability					
Group	Time	ACSA 1 (intra-measure: #1 vs. #2 vs. #3)						ACSA 2 (intra-measure: #1 vs. #2 vs. #3)						ACSA (inter-measure: ACSA1 vs. ACSA2)					
		MEAN	*SD*	*SEM*	-95%	+95%	ICC	MEAN	*SD*	*SEM*	-95%	+95%	ICC	MEAN	*SD*	*SEM*	95%	+95%	ICC
NW	T0	38.1	9.8	1.0	36.2	40.3	99.2	37.6	9.2	1.0	35.7	39.6	99.7	37.9	9.7	1.3	35.6	40.6	99.9
	T1	41.1	11.4	1.2	38.8	43.5	99.9	40.6	11.2	1.2	38.3	43.0	99.9	40.9	11.4	1.5	38.1	44.0	100.0
	T2	37.7	12.6	1.3	35.0	40.3	99.9	37.4	12.6	1.3	34.8	40.0	99.8	37.5	12.6	1.6	34.3	40.9	100.0
SW	T0	41.8	9.8	1.2	39.4	44.3	98.5	41.5	9.6	1.2	39.0	43.9	99.0	41.6	9.8	1.5	38.7	44.8	99.9
	T1	50.2	13.0	1.6	47.0	53.5	99.8	49.9	12.9	1.6	46.7	53.2	99.5	50.1	13.0	2.0	46.1	54.2	99.9
	T2	46.5	12.9	1.6	43.3	49.8	99.9	46.5	12.8	1.6	43.3	49.7	99.7	46.5	12.9	2.0	42.5	50.5	100.0
PLA	T0	51.9	8.8	1.5	48.8	55.1	99.2	51.3	8.8	1.5	48.2	54.4	99.6	51.6	8.8	1.9	47.9	55.6	99.8
	T1	59.8	5.5	0.9	57.9	61.8	98.3	58.7	5.5	1.0	56.8	60.7	98.3	59.3	5.5	1.2	57.1	62.0	99.5
	T2	57.3	6.7	1.2	54.9	59.7	98.5	56.9	7.2	1.2	54.4	59.5	98.8	57.1	6.8	1.4	54.2	60.2	99.8

*Data are expressed as mean ± standard deviation (SD) for each of the measurement of the ACSA (ACSA1, ACSA2) computed from three consecutive measures (#1, #2, #3). Standard error of measurement (SEM), confidence intervals and intra-class correlation coefficients are also reported.*

#### Neuromuscular Properties

##### Isometric and concentric torque

Isometric Maximal Voluntary Contraction (MVC) torque was assessed with an isokinetic dynamometer (Biodex System 2, Biodex Medical Corporation, Shirley, NY, United States). Data were collected and digitized on-line at a rate of 2 kHz (Powerlab 8/35, ADInstruments, Sydney, NSW, Australia). The participants performed three 5 s-MVCs of the KE muscles with at least 60 s of rest between efforts. Visual feedback and verbal encouragements were provided to maximize torque output. Participants sat comfortably on the adjustable dynamometer chair with the hip joint set at 30° (0° = full supine position). The dynamometer lever arm was attached to the right leg by a strap positioned 1–2 cm above the lateral malleolus. Torque was measured at a knee joint angle of 90° (0° = full extension) for KE MVC. For each MVC procedure, the dynamometer axis of rotation was aligned with the joint axis of rotation. Absolute torque was determined as the peak force reached during maximal efforts.

Concentric torque was also measured during three MVCs performed at a velocity of 180°/s over a 110°-range of motion. Peak torque was measured from the best trial and multiplied by contraction velocity (π rad°/s) to calculate the concentric power.

##### Maximal voluntary activation level

Isometric MVCs were superimposed with single magnetic stimulations (Magstim 200^2^, Magstim Co., Whiteland, Dyfed, United Kingdom) to determine the maximal voluntary activation level (VA) of the KE muscles with the twitch interpolation technique (Merton, 1954). Briefly, a superimposed (Qtw_s_) and a potentiated (Qtw_pot_) single twitch were delivered during MVC after the torque had reached a plateau, and 3 s after the cessation of the contraction, respectively. This provided the opportunity to obtain a potentiated mechanical response and hence reduce the variability of VA values. These superimposed and potentiated mechanical amplitudes allowed the quantification of VA as proposed by Merton (1954):

$$\mathrm{VA}=\left[1-{({\mathrm{Qtw}}_{s}\times {\mathrm{Qtw}}_{\mathrm{pot}}}^{-1})\right]\times 100$$

To stimulate the KE muscles, a 70-mm figure-of-eight coil (peak magnetic field strength 2.04 T, stimulation duration 0.1 ms; Magstim Co., Whiteland, Dyfed, United Kingdom) was placed in close proximity to the femoral nerve in the femoral triangle. Stimulations were delivered at supramaximal intensity, i.e., 100% of the stimulator output. This intensity corresponded to 130% of the optimal intensity (i.e., the intensity allowing the complete recruitment of the motor units, as assessed from the plateauing of the evoked torque) [for full details of the stimulation procedures, see Kluka et al. (2015)].

##### Contractile properties

The amplitude of Qtw_pot_, was measured to account for the contractile properties of the KE muscles.

##### Sprinting and jumping performances

Sprinting performances were measured over a 20-m sprint using infrared photoelectric cells (Witty, Microgate Srl, Bolzano, Italy). Cells, positioned at a 1.15-m height, were placed at 20 m from the start line. Participants started in a standing position and ran the 20-m distance as fast as possible. This performance did not include the reaction time. Three trials were performed with a 4-min rest period, and the best performance was retained for subsequent statistical analysis.

Jumping performance was measured during a squat-jump (SJ) using the Optojump system (Optojump, Microgate Srl, Bolzano, Italy). Participants were asked to jump as high as possible with their hands kept on the hips to minimize the contribution of the upper body. The SJ started from a static semisquatting position (90° knee flexion), maintained 1 s; participants were instructed to jump without any preliminary movement. Three trials were performed with a 4-min rest period, and the best performance was retained for subsequent statistical analysis. The average power developed during the SJ was calculated using Lewis formula (Eq. 2):

Power (W)=21.72×mass×√height

### Evaluation of Recovery Kinetics

#### Concentric Power

Concentric torque was measured during three KE MVCs performed at a speed of 180°/s over a 110°-range of motion. Peak torque was measured from the best trial and multiplied by contraction speed (π rad°/s) to calculate the concentric power.

#### Perceived Muscle Soreness

Perceived KE soreness (DOMS) during squatting was evaluated using a visual analog scale consisting in a 100-mm horizontal line with an item at each extremity: from “no pain” to “very, very sore.” Participants were asked to put a vertical mark on the horizontal line to describe the pain experienced during daily locomotion, i.e., level walking, stairs ascent and descent. The distance between the origin of the scale and the vertical mark was used as the pain score.

#### Creatine Kinase Activity

Whole blood CK activity was measured via a fingertip blood sample. A 30 μL sample of blood was collected onto a test strip. The blood sample was analyzed using a colorimetric assay procedure (Reflotron, Roche Diagnostics, Switzerland). Prior to each testing session, quality control (calibration) measurements were undertaken according to the manufacturer recommendations. Capillary blood analyzed using this method displays an intra-assay reliability of <3% coefficient of variation (Howatson and Milak, 2009).

### Statistical Analysis

All the values are reported as mean ± standard deviation (SD). Data were screened for normality of distribution and homogeneity of variances using a Shapiro–Wilk normality test and the Bartlett test, respectively. These two conditions being met, two-way analysis of variance (ANOVA) with repeated measures (group × training status) were performed to assess the effect of training status (T0 vs. T1 vs. T2) and supplementation on the anthropometrical characteristics, neuromuscular properties, sprinting and jumping performances and muscle dimensions. Three-way ANOVAs with repeated measures (session × time × group) were conducted to evaluate the combined effect of time, training status and supplementation on the recovery kinetics of DOMS, CK activity and concentric torque. When the ANOVA revealed significant effects or interactions between factors, a Fisher’s LSD *post hoc* test was applied to test the discrimination between means. For all statistical tests, the limit for statistical significance was set at *P* < 0.05. Statistica 12.0 software (Statsoft, Inc., United States) was used for statistical analysis.

## Results

Baseline characteristics of the different experimental groups are detailed in **Table 1**. Groups did not differ at baseline, excepted for CSA, which was higher in PLA than NW and SW, and fat intake, which was lower in PLA than NW and SW. However, total energy intake and protein intake did not differ between groups.

### Anthropometrical Characteristics and Energy Intake

Energy intake and diet composition did not vary significantly between groups during the training program. Accordingly, body mass remained unchanged during the training period.

### Training Adaptations

#### KE Neuromuscular Properties and Dimensions

**FIGURE 2:**
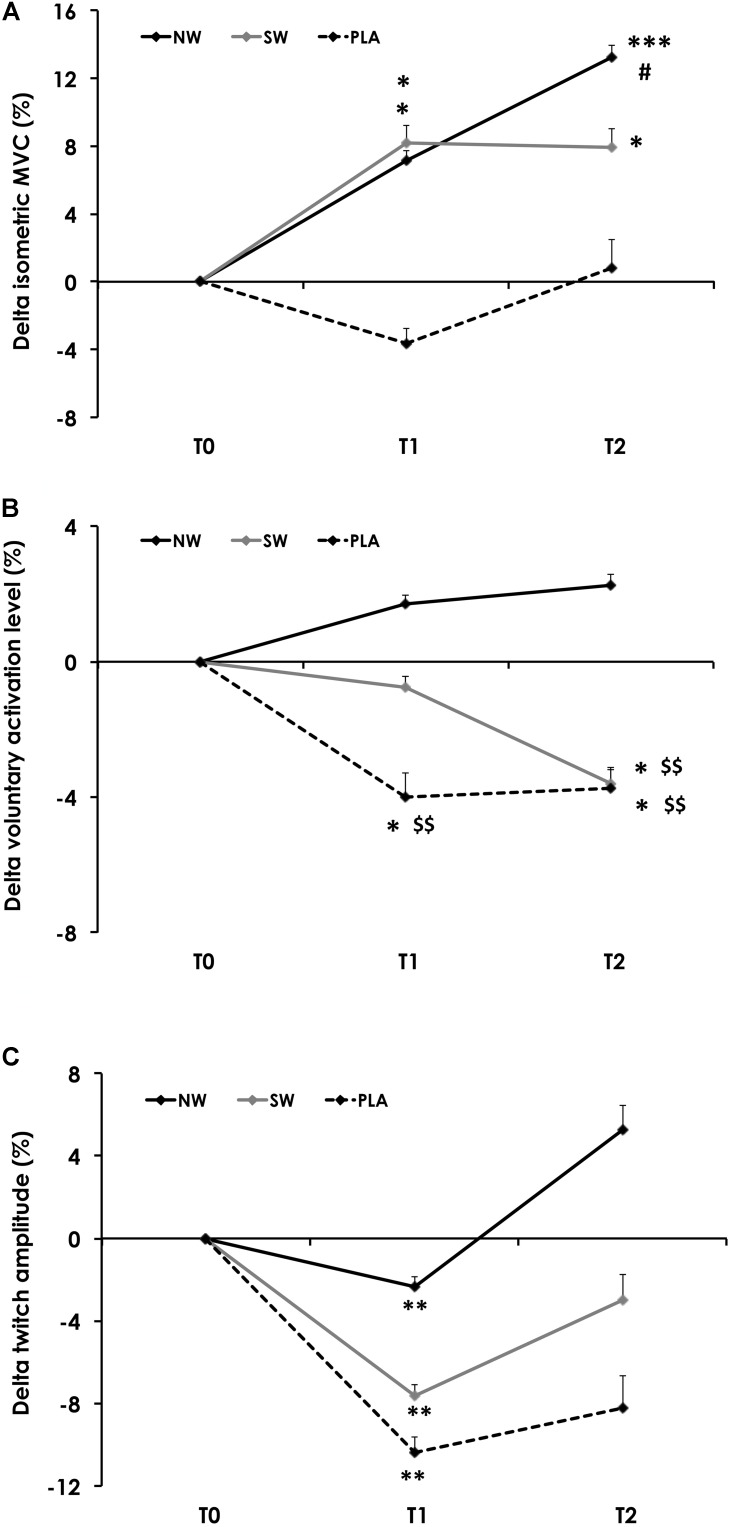
Variation of the isometric maximal voluntary contraction torque (MVC; **A**), voluntary activation level (VA; **B**) and twitch amplitude **(C)** during the training program. Variables were measured before (T0), and after 6 (T1) and 12 weeks (T2) of training. For the sake of clarity, values are expressed as the relative variation from T0. Significantly different from T0: ^∗^*p* < 0.05, ^∗∗^*p* < 0.01, ^∗∗∗^*p* < 0.001. Significantly different from T1: ^#^*p* < 0.05. Significantly different from NW: ^$$^*p* < 0.01.

Isometric MVC force varied as a function of group × training status (*p* < 0.05). MVC force did not change over time in PLA but was increased at T1 and T2 as compared to T0 in the NW and SW groups. NW was the sole group in which MVC force increased between T1 and T2 (**Figure 2A**).

Voluntary activation also varied as a function of group × training status (*p* = 0.01). VA did not change over time in NW but was significantly reduced at T1 and T2 in PLA, and at T2 in SW (**Figure 2B**).

The amplitude of Qtw_pot_ only varied as a function of the training status (*p* < 0.01). On average, Qtw_pot_ amplitude was reduced at T1 (*p* < 0.01) and returned to baseline values at T2 (**Figure 2C**). Similarly, KE CSA only varied as a function of the training status (*p* < 0.001; **Table 4**). On average, KE CSA was increased at T1 (+17.7%; *p* < 0.001) and T2 (+8.3%; *p* < 0.01) as compared to T0. KE CSA also significantly decreased between T1 and T2 (−8%; *p* < 0.01).

Concentric power only varied as a function of the training status (*p* < 0.01). Concentric power was greater at T2 than T0 (597.2 ± 104.6 vs. 551.1 ± 84.4 W, *p* < 0.001) and T1 (597.2 ± 104.6 vs. 527.5 ± 86.8 W, *p* < 0.001).

#### Sprinting and Jumping Performances

Sprinting time decreased significantly during the training period (*p* < 0.05), but did not differ between groups. Sprinting time was significantly reduced at T2 compared to T0 (3.12 ± 0.13 vs. 3.17 ± 0.14 s, respectively, *p* < 0.01). Sprinting time was not significantly different between T0 and T1 (3.17 ± 0.14 vs. 3.15 ± 0.14 s, respectively). Jumping power also varied as a function of the training status, but did not differ between experimental groups. The average power developed during the SJ was not modified between T0 and T1 (903.2 ± 76.4 vs. 906.4 ± 82.8 W, respectively), but was significantly increased at T2 compared to T0 (925.1 ± 87.8 vs. 903.2 ± 76.4 W, respectively, *p* < 0.01).

### Recovery Kinetics

#### Concentric Power

**FIGURE 3:**
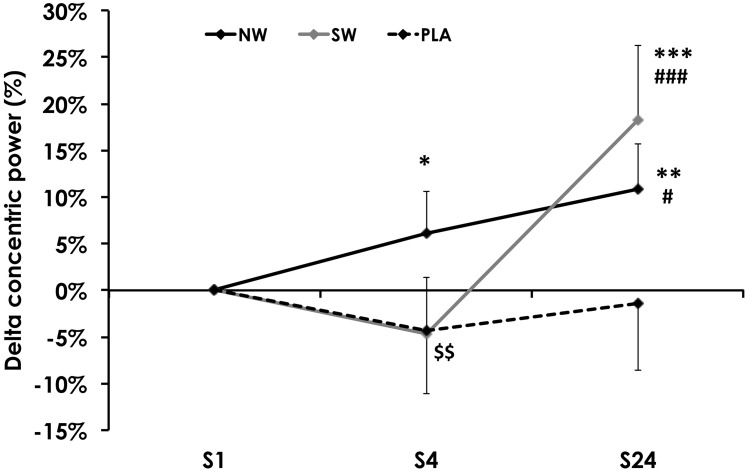
Variation of the concentric power, measured before the 1st (S1, week 1), 4th (S4, week 2), and 24th (S24; week 12) electrostimulation (ES) training session. For the sake of clarity, values are expressed as the relative variation from S1. Significantly different from S1: ^∗^*p* < 0.05, ^∗∗^*p* < 0.01, ^∗∗∗^*p* < 0.001. Significantly different from S4: ^#^*p* < 0.05, ^###^*p* < 0.001. Significantly different from NW: ^$$^*p* < 0.01.

Concentric power, measured before the ES session, varied between groups during the training program (*p* = 0.01). Power increased between S1 and S24 in the NW and SW groups (**Figure 3**). It remained unchanged in PLA. However, concentric power tended to decline between S1 and S4 in the SW group (*p* = 0.06), such that power was significantly reduced in SW compared to NW at S4 (*p* = 0.01).

**FIGURE 4:**
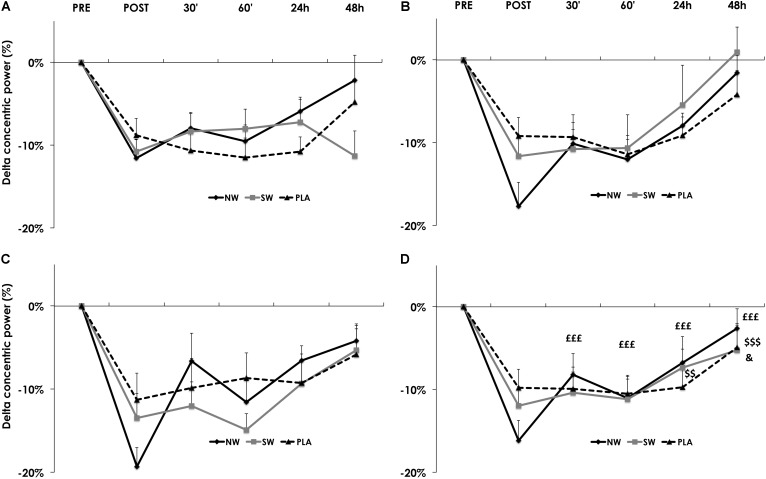
Recovery kinetics of the concentric power, measured before (PRE), after (POST) and 30′, 60′, 24 h, and 48 h after the electrostimulation (ES) training session. Data from S1 **(A)**, S4 **(B)**, S24 **(C)**, and averaged over S1, S4, and S24 **(D)**. For the sake of clarity, values are expressed as the relative variation from PRE. Significantly different from POST in the NW group: ^££££^*p* < 0.001. Significantly different from POST in the SW group: ^$$^*p* < 0.01, ^$$$^*p* < 0.001. Significantly different from POST in the PLA group: ^&^*p* < 0.05.

The recovery kinetics of concentric power after the ES session also varied as a function of group × time (*p* < 0.01). Recovery started at different time points between groups: on average, in the NW group, concentric power started to recover from post-ES session as soon as 30 min after the exercise, whereas recovery started at 24 h after the ES session in the SW group, and 48 h in the PLA group (**Figure 4D**). This behavior appeared progressively over the course of the training program (**Figures 4A–C**).

#### Perceived Muscle Soreness and Creatine Kinase Activity

Creatine kinase and DOMS varied as a function of training status × time (*p* < 0.001), but did not differ between experimental groups. DOMS increased after the ES session and was particularly high at 24 and 48 h (*p* < 0.001). At S4, DOMS was higher at PRE (*p* < 0.01), but lower at 24 and 48 h as compared to S1 (*p* < 0.001). At S24, DOMS was reduced from POST to 48 h after the ES session as compared to S1 (*p* < 0.01).

A similar pattern was observed for CK. CK was increased from baseline at 24 h (1230.8 ± 1038.4 IU/l; *p* < 0.05) and 48 h (2121.5 ± 2435.1 IU/l; *p* < 0.001) after S1. At S4, CK values were significantly higher than S1 values (*p* < 0.001), excepted for 48 h, and peaked 30 min after the ES session (3606.8 ± 5856.0 IU/l). At S24, CK values did not increase after the ES session, such that values were significantly lower than S1 values at 60 min, 24 and 48 h (*p* = 0.05, *p* = 0.01, and *p* < 0.001, respectively). S24 values were also significantly lower than S4 values from PRE to 48 h (*p* < 0.001).

## Discussion

The purpose of the present study was to verify the hypothesis that NW supplementation could promote recovery and training adaptations after an ES training program, as compared to SW. Many of our findings support this hypothesis. Indeed, recovery of concentric power started earlier in the group supplemented with NW. This was associated with a higher concentric power in the early phase of the training program (S4), a preserved VA, and increased isometric force in the second half of the training program (T1–T2). Conversely, in the SW and PLA group, some signs of persistent fatigue were present, such as decreased VA, and this was associated with blunted training adaptations, especially for the isometric force in the second half of the training program.

### Effects of the Training Program

As expected, the ES training program, combined with sprint and plyometrics training, increased KE muscle power. This translated into improvements of running speed over a 20-m sprint and of jumping height. These results are consistent with previous reports showing that an ES-based training program is able to improve neuromuscular properties and performance (Babault et al., 2007; Maffiuletti et al., 2009), especially when the ES-training program is combined with sport-specific movements (Malatesta et al., 2003).

The increased maximal isometric force at T2 was associated with significant KE hypertrophy, which is also consistent with the results of Gondin et al. (2005), who showed that ES training can induce a 6%-hypertrophy of KE CSA after 8 weeks of training. However, the ES program also induced significant muscle damage and fatigue in the first 6 weeks, especially during the 1st week (S1–S4). This was evidenced by significant increases in DOMS, CK activity, and depressed contractile properties at T1. The ∼17% increase in KE CSA at T1 was also certainly partly related to muscle swelling, owing to muscle damage and inflammation. Some groups (PLA and SW) also demonstrated reduced VA, indicative of overreaching or persistent fatigue. Several studies previously demonstrated that ES can induce muscle damage (Nosaka et al., 2011; Jubeau et al., 2012) and overreaching (Zory et al., 2010). It has been proposed that the muscle damage induced by ES is related to the high mechanical stress on the activated muscle fibers due to the specificity of motor unit recruitment (i.e., non-selective, synchronous and spatially fixed manner) (Maffiuletti, 2010). The ability of ES to recruit fast-twitch fibers even at low force levels, certainly also contributes to the large amount of muscle damage and neuromuscular fatigue observed after ES, since fast-twitch fibers are highly fatigable and more prone to muscle damage (Brockett et al., 2002).

These alterations were nevertheless attenuated at the end of the 12-week training program, as evidenced by the significant decrease in CK activity and DOMS at S24. Nevertheless, VA was still reduced in the PLA and SW groups at T2, while it remained unchanged in NW, suggesting that the supplementation significantly influenced recovery kinetics and fatigability during the training program.

### Effects of the Supplementation

The differential effects of the supplements were particularly clear when considering the recovery kinetics of the concentric power, which were accelerated in the participants supplemented with proteins, and especially NW, which promoted short-term recovery. NW is a leucine-rich supplement and interestingly, Reule et al. (2017) recently reported that a leucine-rich amino-acid supplementation, combined to moderate training, improved short-term recovery after a damaging exercise at the end of a training period. Similarly, two studies demonstrated that NW reduced exercise-induced fatigability when combined with a training program in young (Babault et al., 2014) or elderly men (Gryson et al., 2014). In the current study, this faster recovery was associated with better training adaptations, such as the ability to maintain a high VA throughout the training program, which, combined with KE hypertrophy and restored contractile properties, accounted for the increase in the isometric MVC force during the second half of the training program. Concentric power was also higher with NW than SW at the onset (S4) of the training program, suggesting that the NW group better coped with the physical demand of the training program during the first weeks. Rather, VA was depressed at T1 and T2 in PLA and this certainly blunted the beneficial effect of KE hypertrophy at T2, thus preventing any increase of the isometric MVC force in this group. Similarly, the supplementation with SW was not able to prevent the decrease in VA between T1 and T2, which translated into unchanged isometric muscle force, despite KE hypertrophy at T2. Taken together, these results suggest that protein quality can have a significant effect on the recovery and adaptation of the KE neuromuscular properties in response to an ES-program.

However, CK and DOMS were not differentially affected by the supplementation. Although some studies demonstrated reduced CK after a damaging exercise in groups supplemented with proteins (Jackman et al., 2010; Howatson et al., 2012), most of the reports available in the literature show no difference between placebo- and protein-fed groups after exercise (Eddens et al., 2017; Reule et al., 2017), which is consistent with the present results.

The improvement of running speed and jumping power did not differ between groups. This is certainly related to the fact that the combined effect of supplementation and training mainly led to improvements in the KE muscle group, whereas jumping and sprinting tasks require activation of all the muscle groups involved in the extension of the lower-limb (i.e., knee, ankle and hip extensor muscles). Similarly, Babault et al. (2014) did not show any difference in jumping power improvements after training while supplementing young men with NW, casein or a placebo.

The mechanisms underlying the beneficial effect of NW remain to be identified. Unfortunately, the experimental approach used in the current study cannot provide direct evidence. Nevertheless, we can speculate about these effects. As mentioned earlier, NW supplementation has been shown to induce a higher and faster peak value in blood leucine level than with SW consumption after a single bout of strength training (Hamarsland et al., 2017a). Leucine has the potential to stimulate protein synthesis and inhibit proteolysis (Zanchi et al., 2008; Nicastro et al., 2011). This combined influence on synthesis and breakdown could result in a positive net-protein balance, thereby promoting recovery, especially after damaging exercise bouts such as ES. Although this could account for the enhanced recovery 24–48 h after the exercise (which did not differ between groups), it is likely that this potential mechanism cannot fully account for the enhanced short-term recovery. Indeed, recovery within the hour following exercise mainly relates to the restoration of homeostasis (Miller et al., 1987). Interestingly, we recently reported that NW has the potential to stimulate oxidative enzymes (citrate synthase and β-HAD) activities in old rats (Verney et al., 2017). Similarly, Santos et al. (2017) showed that long-term leucine supplementation combined to physical training enhanced citrate synthase activity in young rats, which seems to occur *via* the stimulation of PGC-1α (Costa Junior et al., 2015). Santos et al. (2017) also observed enhanced muscle glycogen content in the trained group supplemented with leucine. This enhanced energy availability combined to improved oxidative metabolism could facilitate the recovery processes, which mainly depend on energy derived from oxidative sources (McMahon and Jenkins, 2002). However, this remains to be demonstrated experimentally. Specifically, it will be important to determine if this potential effect on aerobic metabolism is mediated by leucine, or other mechanisms. The comparable behavior of SW and PLA groups (especially for concentric power), despite a large difference in leucine supplementation, could suggest that other mechanisms are involved.

### Limitations

A few limitations of our study must be noted. First, we chose ES training because it generates recovery issues and potential blunted training adaptations that may be overcome by protein supplementation. Although the findings of the current study may extend to other demanding forms of training, such as eccentric exercise, ES training is not a habitual training approach in the examined population, i.e., recreational sport participants. Additional studies, focusing on more conventional training regimens and other populations, should be conducted to definitively conclude on the practical relevance of NW supplementation in the context of exercise training.

A suboptimal dose (15 g) of protein was given to the participants to avoid a ceiling effect in muscle protein synthesis that would have hidden any potential difference between protein qualities (Bukhari et al., 2015). However, many protein supplements provide 25–30 g per serving. Additional studies examining the dose-effect relationship should be conducted to clarify the practical relevance of NW supplementation. These studies should include biopsies to identify the exact mechanisms underlying the speed-up of the recovery process observed within the first hour after exercise in the current study. Another limitation of the current study was the fact that the spontaneous energy intake of the participants was not fully controlled since they did not receive standard meals, and that none was tested in the fasted state. Added to the fact that the recovery process is highly variable among individuals, this likely induced a large variability in the measurements. Consequently, most of the observed statistical differences were within-group differences, but between-groups differences failed to reach the statistical significance, with the exception of concentric power and VA data.

Taken together, these limitations suggest that additional studies are required to clearly determine the advantage of NW over SW supplementation in the context of physical training.

## Conclusion

The resultsof the present study suggest that NW supplementation could promote short-term recovery and training adaptations after an ES training program, as compared to SW. Although the underlying mechanisms remain to be determined, this beneficial effect of NW could have practical relevance, especially to prevent overreaching during the early phase of training processes, or to promote adaptive responses during high-demanding exercise regimens. Additional studies are nevertheless required to investigate these possibilities, in order to confirm and extend the current findings to other forms of physical training.

## Author Contributions

VM, SR,SG-V, PLB, JB, MB, VV, YC, and NB designed the study. SG-V, EP, CG, AM, CL, FM, YB, VM, and SR collected and analyzed the data. VM, SG-V, SR, PLB, JB, VV, MB, NB, YC, and YB were involved in data interpretation and manuscript preparation. All authors approved the final version of the paper.

## Conflict of Interest Statement

PLR, JB, MB, VV, YC, and NB are employed by Lactalis, which is the funder of the study. The remaining authors declare that the research was conducted in the absence of any commercial or financial relationships that could be construed as a potential conflict of interest.
